# A temporary regulation to manage an impending shortage due to extraordinary prescribing patterns of chloroquines observed during early phase of COVID-19 epidemic

**DOI:** 10.48101/ujms.v128.10033

**Published:** 2023-12-31

**Authors:** Karl-Mikael Kälkner, Anders Sundström, Maria Juhasz Haverinen, Kenneth Nordback, Veronica Arthurson, Björn Zethelius, Rickard Ljung

**Affiliations:** a Swedish Medical Products Agency, Uppsala, Sweden; b Region Stockholm, Stockholm, Sweden

**Keywords:** Temporary regulation, impending shortage, prescribing patterns, chloroquine, hydroxychloroquine, COVID-19 epidemic

## Abstract

**Background:**

Chloroquine and hydroxychloroquine (C/HC) received considerable international media attention due to anticipated treatment effect in COVID-19. This led to increased prescriptions threatening to generate product shortages for patients prescribed within approved indications.

We evaluated effects of a temporary regulation mandating pharmacies to only dispense C/HC prescribed by physicians with defined specialties.

**Methods:**

Data from Region Stockholm, which include 2.4 out of 10 million Sweden’s population, were used. Weekly time trends of prescriptions and requisitions of C/HC by prescriber’s workplace during January to April 2020 were followed.

**Results:**

Numbers of unique individuals with filled prescriptions of chloroquine increased tenfold and of hydroxychloroquine more than threefold from January to March. In the first week of April, filled prescriptions of C/HC dropped. In the later weeks of April, the number of filled prescriptions was back at similar levels as before the SARS-CoV-2 outbreak.

During January and February, specialists in rheumatology accounted for 686 out of all 979 prescriptions dispensed (70.1%) of C/HC. In March, a large proportion of prescriptions dispensed were from specialists not usually prescribing C/HC, and rheumatology accounted for 628 out of all 1,639 prescriptions (38.3%). In April, specialists in rheumatology accounted for 386 out of all 641 prescriptions dispensed (60.0%).

**Conclusion:**

After an observed increase in prescriptions of C/HC, a temporary regulation was introduced on 2nd April 2020 to reduce prescriptions from specialists not usually prescribing C/HC to avoid shortages for patients within approved indications. Subsequently, dispensed prescriptions decreased from April and remained at pre-COVID-19 levels thereafter.

## Background

Chloroquine and hydroxychloroquine (C/HC) received considerable international media attention during the first months of the SARS-CoV-2 pandemic in 2020 as initially anticipated to have effect in COVID-19 treatment (1, 2). In Sweden, physicians can prescribe any approved drug on- or off-label with some rare exceptions. Hydroxychloroquine is used foremost for the treatment of rheumatoid arthritis. However, the use of chloroquine is rather limited.

In March 2020, The Swedish Medical Products Agency (SMPA) received reports from rheumatologists experiencing shortage of hydroxychloroquine for their patients; a rapid increase in dispensed C/HC prescriptions was noted. This occasioned an effort from SMPA to intervene by introducing a temporary regulation for a period of 6 months. As of 2nd April 2020, a temporary regulation (HSLF-FS 2020:11) was in place, mandating that a prescription of chloroquine was only dispensed if the prescribing physician was a specialist in either rheumatology, dermatology-venerology, child and adolescent health, or infectious diseases (3). The goal by this action was to impede the rapid increase of dispensed prescription and regain dispensed prescriptions of chloroquine and hydroxychoroquine to prepandemic levels in order, thus, to secure supply to those patients with an approved indication.

Additionally, information concerning the temporary regulation was published on the SMPA homepage and as a press release through a national wire service, with following publication in trade magazines and newspapers (4–6).

This study evaluated the effects of the temporary regulation by exploring dispensed prescriptions in Sweden and in detail prescribing patterns of C/HC in the Stockholm region.

## Methods

We studied weekly time trends in prescriptions and requisitions of C/HC in the Stockholm Region by prescriber specialty, during January to April 2020. Unique individual prescriptions of either chloroquine or hydroxychloroquine presented weekly starting from the first week in January (Week 1, 30 December 2019 – 5 January 2020) up to the last full week in April (Week 17, 20 April – 26 April). Data from January 2016 to May 2021 were also analyzed. For corresponding drugs, requisitions at hospitals defined as the total number of Defined Daily Doses (DDD) delivered by week were retrieved. Data were acquired from Region Stockholm, the first region in Sweden to be struck with widespread SARS-CoV-2 starting in late February 2020, which include 2.4 out of 10 million Sweden’s population data. All filled prescriptions by residents and all requisitions by hospitals in Region Stockholm were captured. Prescriber’s workplace was used as proxy for prescriber’s specialty.

Data from the National Prescribed Drug Register, governed by the National Board of Health and Welfare, concerning the number of unique patients with dispensed C/HC were used for the study of monthly time trends during 2020.

## Results

**Figure 1:**
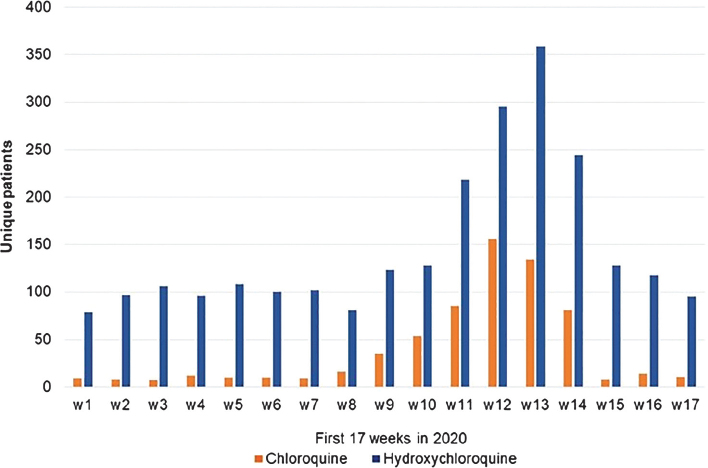
Numbers of unique patients dispensed filled prescriptions of chloroquine or hydroxychloroquine during weeks 1 to 17 in 2020, that is, January–April 2020, in the Stockholm Region.

In Region Stockholm, the number of unique individuals with filled prescriptions of chloroquine increased tenfold, and that of hydroxychloroquine more than threefold from early January to last weeks of March (Figure 1). During the first week of April (week 14), filled prescriptions of C/HC dropped, and in the later weeks of April, the number of filled prescriptions was back at similar level to that before the SARS-CoV-2 outbreak. This contrasts to the previous years characterized by an even distribution through the years with a small increase in numbers of filled prescriptions noted; for the corresponding years 2016 to 2019, the average numbers (95% confidence interval) were 397 (382–411), 431 (417–445), 431 (419–444), and 459 (446–472).

Requisitions expressed as DDD of chloroquine increased to more than 35,000 in March from being almost zero in January and February, and DDD of hydroxychloroquine increased to nearly 2,000 in the last week of March from being less than 100 in January and February. In the later weeks of April, the requisition of C/HC dropped to nearly zero, similar to levels before the SARS-CoV-2 outbreak.

During January and February (numbers added together), specialists in rheumatology accounted for 686 out of all 979 prescriptions dispensed (70.1%) of chloroquine or hydroxychloroquine, followed by general practitioners and specialists in dermatology-venerology (Table 1). In March, a large proportion of prescriptions dispensed were from specialists not usually prescribing these drugs, and rheumatology accounted for 628 out of all 1,639 prescriptions (38.3%). In April, specialists in rheumatology accounted for 386 out of all 641 prescriptions dispensed (60.0%).

**Table 1:** Number of filled prescriptions of chloroquine and hydroxychloroquine by prescriber’s specialty, Stockholm Region, January–April 2020.

Prescriber’s specialty^a^	January	February	March	April
	*N*	*N*	*N*	*N*
Rheumatology	351	335	628	386
Dermatology-venerology	16	8	43	29
Child and adolescent medicine	2	2	21	5
Infections disease	0	0	0	0
Other	121	144	947	221
Total	490	489	1,639	641

a Prescriber’s workplace used as proxy for specialty.

**Figure 2:**
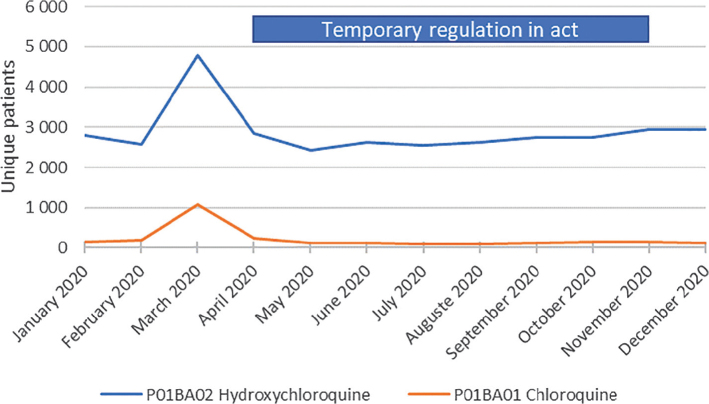
Numbers of unique patients monthly dispensed filled prescriptions of chloroquine or hydroxychloroquine during January to December in 2020 in Sweden. After the temporary regulation entered into force, a rapid decline of dispenses was observed during April 2020. Data source: The National Prescribed Drug Register.

Using data from The National Prescribed Drug Register, it was observed that between February and March, the number of unique patients with dispensed chloroquine and hydroxychlorquine increased from 183 to 1,073 (486%) and 2,575 to 4,793 (86%), respectively. From May onward, the number of unique patients was similar to previous levels of prescriptions, and no differences were noted when the regulation was terminated (Figure 2).

## Limitations of the study

This is an observational study using prescribing information in available data sources and does not include data of other explanatory factors that affect prescription such as general state of knowledge, knowledge of the individual prescriber, media attention, prices, availability, national regulation, etcetera. So, differentiating the exact role of this temporary regulation has not been possible.

The data of prescriber specialty derive from Stockholm Region, which has more specialists per 100,000 inhabitants than in average in Sweden, and thereby, it could be expected that changes in prescribing patterns are highly marked in Stockholm than in average in Sweden.

The working place has been used as a substitute for specialty. This is accompanied by some uncertainties such as not all prescribers working at a department of rheumatology are specialists. Additionally, there are prescribers who have multiple specialties that could work outside the four specified specialties, for example, a combination of internal medicine and rheumatology working at a medicine clinic. Furthermore, there are workplaces that are classified as ‘Other’ since they include several specialties. Nevertheless, the uncertainties are judged to be the same before and after the intervention, and the group ‘Other’ largely contributed to the increase.

## Discussion

A use of a temporary regulation is one way for a regulatory authority to intervene; however, the toolbox for national authorities to regulate prescribing differs depending on national legislations. Measures could include repetition of basic information such as approved indication and possible side effects. While escalating the imperative, different forms of authority recommendation could be implemented such as restricted dispensing to on-label treatment or restriction of funding to on-label treatment. Examples of different strategies to control the dispensing of hydroxychloroquine during the pandemic have been reported from several countries.

Regulatory authorities often use communications as method to inform about updated knowledge and repeat information when it is deemed necessary. When assessing the effects of provided information, there are several factors that influence the outcome, such as the method of distribution, the knowledge of the medicine alert, perceptions of alerts in general, and attitudes and concerns regarding medicine alerts. The effect of provided information is not commonly reported. Nevertheless, the timing of information and following changes in prescribing patterns have been reported.

In the United States, the Food and Drug Administration (FDA) approved an emergency-of-use authorization for C/HC for the treatment of COVID-19 on 28 March 2020 and then issued a statement of caution on 24 April 2020, followed by the revoke of the emergency-of-use authorization on 15 June 2020. From February 2020 to March 2020, Shehab et al. observed an increase in number of patients receiving dispensed hydroxychloroquine prescriptions from 367,346 to 683,999 (86.2%), and when using data from the IQVIA Total Patient Tracker, it was observed that the number of patients receiving dispensed chloroquine increased from 2,346 to 6,066 (158.6%) (7). The Centers for Disease Control and Prevention (CDC) compared outpatient retail pharmacy transaction data in the United States for the first half of 2019 with the first half of 2020. During March and April 2020, monthly C/HC outpatient prescribing was higher than it was during the previous year. However, in May and June 2020, Bull-Otterson et al. observed a decrease in numbers of new prescriptions in total and new prescriptions from non-routine prescribing specialties, approaching the levels of 2019 (8). Cause and effect of the regulatory action is difficult to conclude, but the prescription rate of C/HC went back to baseline levels after 2 months.

The outcome of mandatory regulatory actions related to C/HC has been published.

In Australia, on 24 March 2020, due to concerns of off-label prescribing of hydroxychloroquine, the Therapeutic Goods Administration (TGA) limited those who could initiate therapy to those with a relevant medical specialty as per the Medical Board list: dermatology, intensive care medicine, pediatrics and child health, physician, and emergency medicine. Additionally, in May 2020, the Department of Health and Aged Care changed the Pharmaceutical Benefits Scheme list to reflect the changes made by the TGA. Hereafter, prescribers had to ensure that patients met the relevant restriction criteria in order to the TGA to subsidize hydroxychloroquine. A large spike in hydroxychloroquine dispensing was observed in March 2020, and the authors concluded that the greatest relative increase was related to new users, and more likely to have been prescribed by a General Practitioner rather than a specialist (9).

On 24 March 2020, the regulatory authorities in New Zealand restrict the funded supply to the registered indications only: active rheumatoid arthritis, systemic and discoid lupus erythematosus, malaria treatment, and malaria suppression (10). Subsequently, a decrease in filled prescriptions for hydroxychloroquine for patients with no previous dispensing records for hydroxychloroquine was noted within a couple of weeks (11).

Both Australia and New Zealand have national legislations that regulate subsidizing pharmaceutical cost for patients at pharmacy levels and have operational supporting system in place. In that regard, there is in place a regulatory measure that could be used in an acute upcoming situation with pending shortage.

In Sweden, the national legislation permits a possibility for SMPA to restrict dispensing prescriptions at pharmacies. There are five cases in action involving restriction concerning prescriptions of substances that are classified as narcotics in treatment of Attention Deficit Hyperactivity Disorder (ADHD), substances approved for maintenance treatment in opioid dependence, the substances isotretinoin and alitretinoin for acne treatment, and the substances mifepristone to end a pregnancy and esketamine for nasal administration in cases of grave depressive illness. Hence, the pharmacies already had a routine in place to manage similar measures of restrictions, which contributed to the successful outcome in this case with the temporary regulation of C/HC. Parallel to the temporary regulation, alerts were published regarding the risk of C/HC concerning increased risk of heart problems, including cardiac arrhythmias and cardiac arrest (European Medical Agency dated 23 April 2020 and 29 May 2020). Additionally, on 5 June 2020, the RECOVERY trial decided to stop enrolling participants to the hydroxychloroquine treatment arm as no clinical benefit from the use of hydroxychloroquine in hospitalized patients with COVID-19 had been demonstrated (12, 13).

During late spring/early summer in 2020, the knowledge about the risks of C/HC was actualized, and the growing experience of limited effect in treating and the prevention of COVID-19 was evident. Whether this knowledge alone would have been sufficient to prevent an ongoing high rate of prescription in Sweden has not been possible to study. But experiences from the United States could be interpreted as that the rapid increase in the prescriptions of C/HC was only temporary, and that it was due to a mix of patients who were already on the medication getting extra supplies and of persons being prescribed the medicine inappropriately in the despair of COVID-19 disease. These inappropriate prescriptions decreased over time as knowledge rapidly evolved.

In Germany, the Bundesinstitut für Arzneimittel and Medizinprodukte reported a shortage of supply of hydroxychloroquine sulfate 200 mg tablets from April to August 2020, and on 3 April 2020, they issued a recommendation that hydroxychloroquine should only be prescribed on an outpatient basis if an approved indication is specified, and that a prescription should be limited to a maximum of 100 tablets of 200 mg each (14). In a drug utilization study analyzing drug prescriptions in the database of the German Institute for Drug Use Evaluation covering 88% of the Germany’s population, an increase of 110% in packages per week was noted in March 2020 for hydroxychloroquine, followed by a decrease in April 2020, and the authors concluded that existence of misinformation, speculations of effect, and shortages of supply influenced drug prescribing (15).

Altogether, the regulatory authorities should have a preparedness plan with available measures to act rapidly in situations with pending shortage to secure the supply to those patients in most need treated within approved indications.

## Conclusion

On 2 April 2020, the SMPA decided on a 6-month temporary regulation restricting the pharmacies dispensing of chloroquine or hydroxychloroquine to prescribers with relevant specialties. This was done in order to reduce inappropriate prescriptions and to thereby avoid shortage for those patients within approved indications. Subsequently, the number of dispensed prescriptions decreased from April and remained at previous levels thereafter.

This serves as an example of how to manage an impending shortcoming situation that, in this case, was caused by assumptions of possible therapeutic effects in a precarious situation before required knowledge had been obtained.
